# Career pathways and professional backgrounds associated with NBA head coaching status: evidence from the NBA, G-League, and NCAA

**DOI:** 10.3389/fspor.2026.1820197

**Published:** 2026-07-02

**Authors:** Ágoston Nagy, Botond Ágoston Nagy, Szilvia Borbély, Benedek Ágost Nagy

**Affiliations:** 1 Institute of Sport Sciences, University of Debrecen, Debrecen, Hungary; 2 School of Doctoral Studies, Hungarian University of Sports Science, Budapest, Hungary; 3 Institute of Physical Education and Sports Science, Nyíregyháza University, Nyíregyháza, Hungary

**Keywords:** career pathways, coaching, coaching development, NBA, professional background

## Abstract

**Introduction:**

This study examined demographic, playing-career, and coaching-career characteristics associated with NBA head coaching status by comparing NBA, NBA G-League, and NCAA Division I head coaches.

**Methods:**

Data were collected during the 2020/21 NBA preseason, a period following the COVID-19-affected 2019/20 season, from publicly available biographical and professional sources. The final sample included 76 head coaches: 30 NBA head coaches, 30 randomly selected NCAA Division I head coaches, and 16 NBA G-League head coaches. Variables were categorized into demographic/background, playing-career, and coaching-career characteristics. Descriptive statistics, chi-square tests, one-way ANOVA, and hierarchical binary logistic regression analyses were conducted.

**Results:**

Significant differences were identified across coaching environments in age, NBA playing experience, and international playing experience. NCAA coaches were the oldest group, while NBA G-League coaches were the youngest. The strongest between-league difference was observed for previous NBA assistant coaching experience. In the final hierarchical binary logistic regression model, previous NBA assistant coaching experience showed the strongest association with NBA head coaching status, while NBA playing experience also remained significant. Age and White race/ethnicity were not significant predictors.

**Discussion:**

The findings suggest that NBA head coaching status is more closely associated with NBA-specific professional experience and organizational exposure than with demographic or formal educational characteristics alone.

## 1 Introduction

Coaching job is a multifaceted job in every sports. In major professional leagues, particularly in the NBA, head coaching is a complex leadership role that requires continuous learning, risk-taking, and decision-making under pressure. It entails a high degree of developmental pressure and competitive confrontation under conditions shaped by the characteristics and actions of the opponent. They must perform tasks that span multiple disciplines, including psychology, pedagogy, and training theory, among others. Coaching effectiveness is strongly influenced by how coaches communicate, teach, and manage interpersonal relationships within the team environment. It is not just what they do that matters, but also how they do it. Every player and staff member has unique needs. Some respond better to praise and others to criticism. Coaches' supportive and negative reactions directly affect athletes' motivation, self-confidence and anxiety. Based on the scientific results, the coaches' supportive behavior showed a positive correlation with the athletes' self-confidence and intrinsic motivation, while negative reactions increased the athletes' anxiety levels (1). Great coaches are not just leaders, but also mentors to their players and assistant coaches on and off the basketball court. The approaches to mentorship can vary. Among the 11 different mentor roles, the most effective were friend, acceptor, and challenger (2).

**Figure 1:**
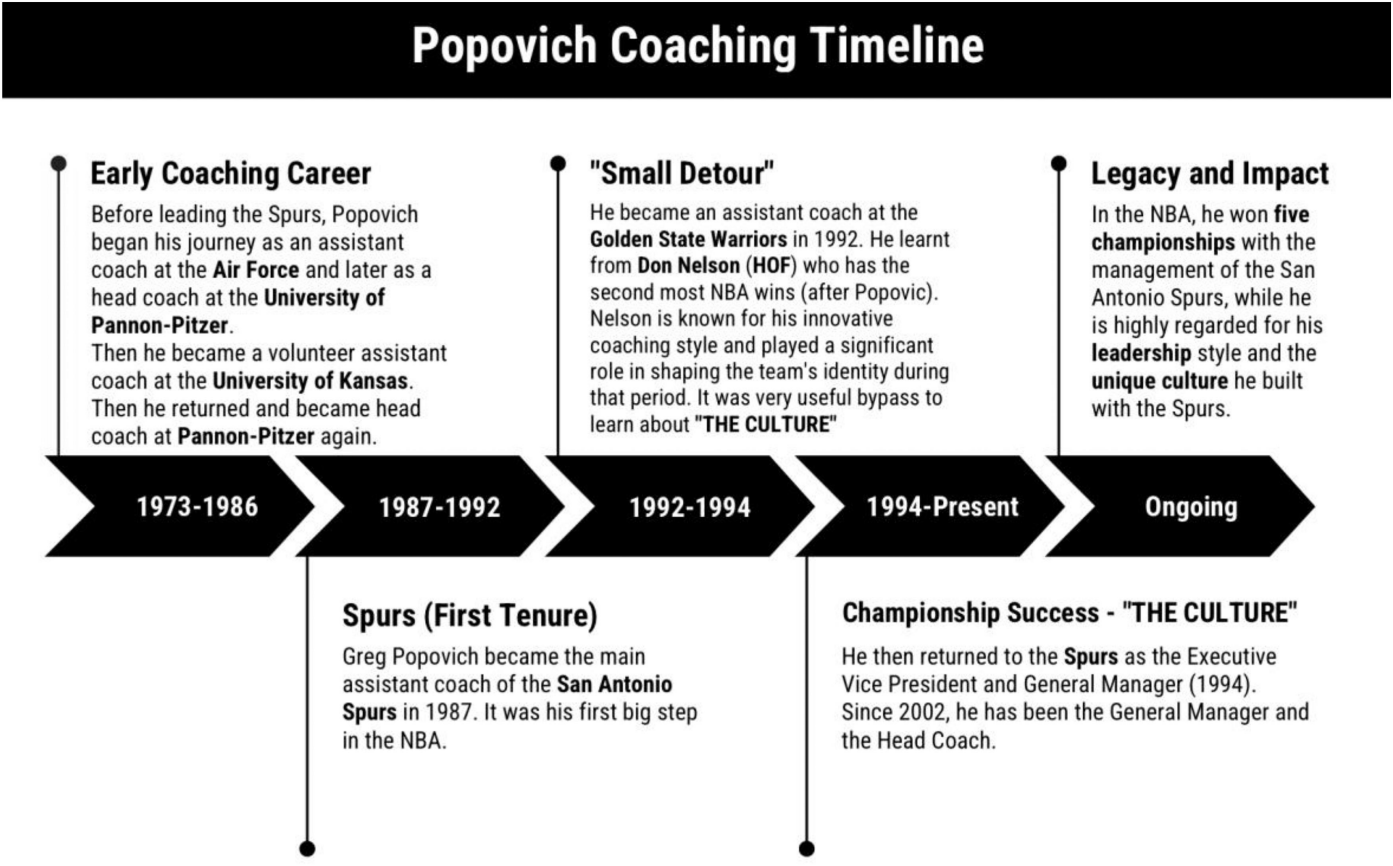
The remarkable journey of Gregg Popovich throughout his career milestones with various teams and roles. Source: Original figure created by the authors using publicly available data from official NBA and team websites.

They constantly make decisions before, during and even after the games. They try to balance the interests of the players and the team. However, it should not be forgotten that in the NBA, winning and making profit are the main goal of the teams and league. The high-performance environment of the NBA creates substantial occupational pressure and job insecurity for head coaches. Even if the career of NBA coaches - in terms of time spent with a team - has never been so stable. In the 2019/2020 season, head coaches spent an average of 303.5 games with the same team. In other words, it is expected that they will be fired after almost four seasons on average. This is the highest number since the NBA's inception (1946) (3). Gregg Popovich spent the longest tenure as a head coach - 25 years - at the same team (Figure 1). If we exclude his data, the value is still 242.0 games. The number of coaching changes during the season has not changed in the last forty years (3).

Over the past 20 years, the NBA has had the most coaching changes of the four North American Major League sports (NBA, NFL, NHL and MLB). On average, coaching changes occur every 2.4 seasons in the NBA, compared to 2.6 in the NHL, 3.1 in the MLB, and 3.4 in the NFL, meaning that basketball coaches have less than 2.5 seasons to be successful. Teams that change coaches less often have a higher win-loss ratio. Gregg Popovich's tenure as a head coach is the longest among coaches in the four leagues (Figure 1), with a 68.6 winning percentage. Several examples illustrate the instability and mobility of NBA coaching careers. Since 2000, some NBA franchises have changed head coaches frequently, while several coaches have also led multiple teams. Overall, NBA coaches appear to face substantial job insecurity, but they may also have greater opportunities to be rehired by another franchise compared with coaches in other major North American professional leagues (4).

Coaching stability may contribute to organizational success, as long-term NBA coaches have represented only 22% of all coaches since 1946, but accounted for 28% of playoff coaches and 35% of NBA titles (5). However, experience alone does not necessarily make leadership performance predictable (6).

A study by the Sports Economist examined the impact of coaching changes in the American college football. According to Adler et al. (7), for poorly performing teams, switching the coaches resulted in some improvement compared to those that did not switch. However, in the mid-tier teams, the performance declined following a coaching change compared to those that maintained long-term coaching stability.

In order to achieve faster success, it is more effective to change the player base. Audas et al. (8) and Berri et al. (9) examined the role of coaches in individual player performance. While some highly rated coaches deserve credit, there are coaches who owe their success to their talented teams. On the other hand, some coaches with average results improved the individual performance of their players with significantly greater success. Most head coaches do not have a decisive impact on player performance.

There are several ways and stages of coach development. How they start this journey can also be very different. Some people start as video analysts, as a player development coach or as an assistant coach. No one begins their career as a head coach. This is a process in which a playing experience can be an advantage. To become a head coach/manager in the major professional sports, individuals often start as players in that sport and at that level (10). Playing experience can be highly beneficial in some cases as it allows coaches to better understand players' emotions and competitive stress and team dynamics (11). Moreover, players, especially guards turned coaches make better strategic decisions (12). A certain level of experience as a player may be necessary, but it is not sufficient on its own to become a top-level coach (13). The knowledge acquired in this way can also be learned through other methods (14). Coaches may develop beyond their own playing experience through coaching clinics, mentoring, role models, and interaction with other coaches. Mentor coaches are important for younger coaches to gain knowledge (15). Over time, this method is completely replaced by interactions with fellow coaches.

Throughout the learning process in addition to professional knowledge, coaches also develop leadership skills in this process (16–20). Leadership is not an innate ability, but the result of a long learning process, at the end of which they become excellent leaders and coaches. This is judged based on their behavior, actions and, of course, effectiveness (21). Many great coaches started their careers as players, assistant coaches, video analysts, or in other positions around the team. Working in various coaching positions provides the opportunity to gain the necessary coaching experience and knowledge. A coaching career takes place step by step, during which individuals learn from useful and often painful experiences and then advancing by recognizing and developing their own strengths and weaknesses.

Although previous research has examined coaching leadership, mentoring, and coaching development processes, much less attention has been paid to the professional and demographic career paths that are associated with NBA head coaching positions. In particular, little research has compared NBA, NCAA, and NBA G-League coaches to better understand how different career paths may contribute to advancement opportunities within elite basketball organizations. Given the highly competitive and unstable nature of the professional basketball coaching career, understanding which professional experiences and demographic characteristics may be associated with the NBA head coaching positions can provide valuable insight into coaching career development and organizational selection patterns.

Despite the growing literature on coaching development, limited research has examined demographic and professional career pathways associated with NBA head coaching status across different basketball systems.

Bandura's (22) framework may provide a conceptual perspective for understanding how accumulated professional experiences contribute to coaching development.

Coaching careers may develop through multiple professional pathways, where accumulated experience, organizational exposure, and leadership opportunities contribute to career advancement. This professional knowledge is crucial. The NBA serves as the benchmark league for basketball, attracting the most valuable players in the sport.

The aim of the study was to examine whether demographic and professional background variables are associated with NBA head coaching status across the NBA, NCAA, and NBA G-League.

H1: Coaches with prior NBA assistant coaching experience are more likely to attain NBA head coaching positions.

H2: Demographic characteristics, particularly age, are associated with NBA head coaching status.

H3: Coaching career trajectories significantly differ across NBA, NCAA, and G-League coaching environments.

## 2 Material and methods

The study was conducted using data from the 2020/21 NBA preseason, a period that followed the shortened 2019/20 season affected by the COVID-19 pandemic and preceded an unusually late start to the subsequent NBA season. The sample included head coaches from three major basketball contexts in the United States: the NBA, the NBA G-League, and NCAA Division I. All 30 NBA head coaches were included. In addition, 16 NBA G-League head coaches and 30 randomly selected NCAA Division I head coaches were analyzed. The final sample therefore consisted of 76 coaches. The NCAA Division I coaches were selected randomly to provide a more broadly reflective comparison group of the NCAA Division I coaching population and to avoid limiting the comparison to a performance-based subgroup.

First, we obtained data from the official league websites and from the coaches' professional biographies. These have been supplemented with information found on the teams' official websites. We cross-checked the data using the coaches' personal websites and other online sources.

The size of the G-League sample was limited by the specific circumstances of the 2020/21 season. Due to the COVID-19 pandemic, the G-League season was organized in a bubble system, and not all G-League teams participated under the usual regular season conditions. Only 18 teams took part. At the time of data collection, the start of the next season for several G-League teams and the identity of the head coach had not yet been officially confirmed. Only those G-League head coaches for whom publicly available and verifiable information was available at the time of data collection could be included in the sample. Although 18 NBA G-League coaches were initially considered, two cases were excluded during data verification because complete and reliable data were not available for all variables included in the analysis. As a result, the final NBA G-League subsample consisted of 16 coaches.

The collected information was organized into three categories. The first category included demographic and background variables, such as age, education, nationality, race/ethnicity, and family-related basketball background. This category contained 11 coded variables. The second category focused on playing career characteristics and included 10 variables related to previous basketball playing experience, such as NCAA, NBA, G-League, and international playing history. The third category described the coaches' professional career pathways and included 27 variables related to coaching experience, previous coaching roles, organizational background, and career progression.

The study was based exclusively on publicly available biographical and professional information, therefore no direct contact with coaches was made and no questionnaire, interview, or self-report survey was administered. As a result, no informed consent was required. The dataset did not include private, sensitive, or non-public personal information. Data were coded for research purposes only, and all analyses were conducted at the aggregate level. Because the study used only publicly available professional and biographical information and did not involve direct interaction with participants, intervention, surveys, interviews, private data collection, or sensitive personal data, formal ethical approval and informed consent were not required. All data were analyzed at the aggregate level and used exclusively for research purposes.

Following data entry and data cleaning, statistical analyses were conducted to examine demographic differences, professional background characteristics, and factors associated with NBA head coaching status. Descriptive statistics were calculated for all relevant variables. Continuous variables were reported as means and standard deviations, while categorical variables were summarized using frequencies and percentages. Group differences among NBA, NCAA, and G-League coaches were examined using cross-tabulation analyses and chi-square tests for categorical variables. For age, which was treated as a continuous variable, mean differences among NBA, NCAA, and NBA G-League coaches were examined using one-way analysis of variance (ANOVA). This analysis was used to assess whether the three coaching environments differed in terms of coaches' average age. The effect size for the ANOVA was reported using eta squared (η^2^). Cramer's V was reported as the effect size for chi-square analyses of categorical variables.

To examine factors associated with NBA head coaching status, binary logistic regression analysis was conducted. The dependent variable was NBA head coaching status, coded as 1 for NBA head coaches and 0 for NCAA or G-League head coaches. Predictor variables included selected demographic and professional background characteristics, such as age, NBA playing experience, NCAA playing experience, international playing experience, and previous NBA assistant coaching experience. Model results were reported using unstandardized regression coefficients, standard errors, Wald statistics, *p*-values, odds ratios, and 95% confidence intervals. Model fit was evaluated using the likelihood-ratio chi-square test, Nagelkerke R^2^, the Hosmer–Lemeshow goodness-of-fit test, and classification accuracy. Statistical significance was set at *p* < 0.05. Given the relatively small analytic sample, particularly for multivariable regression, the number of predictors included in the final model was deliberately restricted to reduce the risk of overfitting and to improve model interpretability. Predictors were selected based on theoretical relevance, reviewer feedback, and their relevance to the study aim. Therefore, the hierarchical binary logistic regression was structured in a limited number of steps, beginning with demographic variables and then adding NBA-related playing and coaching experience.

## 3 Results

### 3.1 Sample characteristics and descriptive statistics

The cleaned dataset contained 76 coaches: 30 NBA head coaches, 30 randomly selected NCAA Division I head coaches, and 16 NBA G-League head coaches. Table 1 presents the descriptive statistics and between-league comparison. Age differed significantly across the three coaching environments, F(2, 73) = 23.06, *p* < 0.001, eta^2^ = 0.387. NCAA coaches were the oldest group (M = 56.87, SD = 8.79), followed by NBA coaches (M = 50.50, SD = 9.30) and NBA G-League coaches (M = 38.62, SD = 7.05).

**Table 1:** Descriptive statistics and between-league comparison.

Variable	NBA	NCAA	G-league	Test statistic	*p*	Effect size (*η*^2^/Cramer's V)
Age, years	50.50 (9.30)	56.87 (8.79)	38.62 (7.05)	F(2, 73) = 23.06	< 0.001	eta^2^ = 0.387
White race	21 (70%)	26 (86.7%)	13 (81.2%)	*χ*^2^ (2) = 2.57	0.276	Cramer's V = 0.184
African American race	7 (23.3%)	4 (13.3%)	4 (25.0%)	χ^2^ (2) = 1.30	0.522	Cramer's V = 0.131
University degree	29 (96.7%)	29 (96.7%)	14 (87.5%)	χ^2^ (2) = 2.13	0.345	Cramer's V = 0.167
Master's degree	3 (10%)	13 (43.3%)	1 (6.2%)	χ^2^ (2) = 12.63	0.002	Cramer's V = 0. 408
Sport/PE degree	4 (13.3%)	10 (33.3%)	3 (18.8%)	χ^2^ (2) = 3.61	0.165	Cramer's V = 0. 218
Coach in family	7 (23.3%)	6 (20%)	2 (12.5%)	χ^2^ (2) = 0.78	0.679	Cramer's V = 0.101
NBA player in family	3 (10%)	0 (0%)	1 (6.2%)	χ^2^ (2) = 3.05	0.218	Cramer's V = 0.200
NBA worker in family	5 (16.7%)	1 (3.3%)	3 (18.8%)	χ^2^ (2) = 3.48	0.175	Cramer's V = 0.214
NCAA playing experience	28 (93.3%)	26 (86.7%)	15 (93.8%)	χ^2^ (2) = 1.01	0.604	Cramer's V = 0.115
NBA playing experience	9 (30%)	1 (3.3%)	3 (18.8%)	χ^2^ (2) = 7.561	0.023	Cramer's V = 0.315
International playing experience	7 (23.3%)	4 (13.3%)	8 (50%)	χ^2^ (2) = 7.556	0.023	Cramer's V = 0.315
NBA assistant coaching experience	25 (83.3%)	5 (16.7%)	6 (37.5%)	χ^2^ (2) = 27.53	< 0.001	Cramer's V = 0.602
NCAA head coaching experience	7 (23.3%)	30 (100%)	1 (6.2%)	χ^2^ (2) = 50.78	< 0.001	Cramer's V = 0.817
G-league head coaching experience	5 (16.7%)	1 (3.3%)	16 (100%)	χ^2^ (2) = 51.04	< 0.001	Cramer's V = 0.820

*η*^2^ was used as the effect size for continuous variables analyzed with ANOVA, whereas Cramer's V was used for categorical variables analyzed with chi-square tests.

Race/ethnicity distribution: no statistically significant between league difference was detected in the present sample.

Educational variables showed mixed results. University degree status did not differ significantly across leagues. However, master's degree status differed significantly by league, χ^2^(2) = 12.63, *p* = 0.002, Cramer's V = 0.408, with NCAA coaches showing the highest proportion of master's degrees (Table 1). Sport or physical education degree status did not differ significantly across leagues.

### 3.2 Playing and coaching career background

NCAA playing experience was common across all three groups and did not differ significantly by league. NBA playing experience differed significantly across leagues, χ^2^(2) = 7.561, *p* = 0.023, Cramer's V = 0.315. NBA head coaches showed the highest proportion of prior NBA playing experience (30.0%), followed by NBA G-League coaches (18.8%) and NCAA coaches (3.3%) (Table 1).

International playing experience also differed significantly by league, χ^2^(2) = 7.556, *p* = 0.023, Cramer's V = 0.315., with the highest proportion observed among NBA G-League coaches (50.0%).

The strongest between-league difference among the professional background variables was observed for NBA assistant coaching experience, χ^2^(2) = 27.53, *p* < 0.001, Cramer's V = 0.602. NBA head coaches were much more likely to have previous NBA assistant coaching experience (83.3%) than NCAA coaches (16.7%) or NBA G-League coaches (37.5%) (Table 1).

NCAA head coaching experience and NBA G-League head coaching experience also differed significantly across groups, but these results should be interpreted cautiously because these variables partly reflect the structural definition of the compared coaching environments.

### 3.3 Factors associated with NBA head coaching status

A hierarchical binary logistic regression was used to examine whether demographic and professional background variables were associated with current NBA head coaching status. The dependent variable was NBA head coaching status, coded as 1 for NBA head coaches and 0 for NCAA or NBA G-League head coaches. Table 2 shows the hierarchical binary logistic regression models examining factors associated with NBA head coaching status.

**Table 2:** Hierarchical binary logistic regression models examining factors associated with NBA head coaching status.

Model	Predictor	B	SE	Wald	*p*	OR	95 CI for OR
M1	Constant	0.168	1.198	0.02	0.888		
M1	Age	0.002	0.022	0.01	0.939	1.00	[0.96, 1.05]
M1	White race	−0.873	0.573	2.32	0.128	0.42	[0.14, 1.28]
M2	Constant	−0.335	1.256	0.07	0.790		
M2	Age	0.005	0.023	0.06	0.813	1.01	[0.96, 1.05]
M2	White race	−0.799	0.596	1.79	0.181	0.45	[0.14, 1.45]
M2	NBA playing experience	1.464	0.668	4.80	0.029	4.32	[1.17, 16.02]
M3	Constant	−3.146	1.752	3.22	0.073		
M3	Age	0.016	0.029	0.31	0.581	1.02	[0.96, 1.07]
M3	White race	−0.215	0.729	0.09	0.768	0.81	[0.19, 3.37]
M3	NBA playing experience	2.151	0.886	5.89	0.015	8.59	[1.51, 48.83]
M3	NBA assistant experience	3.068	0.710	18.68	< 0.001	21.49	[5.35, 86.40]

OR, odds ratio; CI, confidence interval; M1; age and White race/ethnicity; M2, M1 + NBA playing experience; M3, M2 + NBA assistant coaching experience. The dependent variable was coded as 1 = NBA head coach and 0 = NCAA or NBA G-League head coach.

Model 1, which included age and White race/ethnicity, was not statistically significant, χ^2^(2) = 2.35, *p* = 0.309, Nagelkerke R^2^ = 0.041. After NBA playing experience was added in Model 2, the model approached statistical significance, χ^2^(3) = 7.54, *p* = 0.056, Nagelkerke R^2^ = 0.128. NBA playing experience was significantly associated with higher odds of NBA head coaching status in Model 2 [OR = 4.32, 95% CI (1.17, 16.02), *p* = 0.029].

The final model (M3), which added previous NBA assistant coaching experience, was statistically significant, χ^2^(4) = 34.76, *p* < 0.001, Nagelkerke R^2^ = 0.497. In the final model, previous NBA assistant coaching experience showed the strongest independent association with NBA head coaching status [OR = 21.49, 95% CI (5.35, 86.40), *p* < 0.001]. NBA playing experience also remained significantly associated with NBA head coaching status [OR = 8.59, 95% CI (1.51, 48.83), *p* = 0.015]. Age and White race/ethnicity were not statistically significantly associated with NBA head coaching status in the final model.

The final model correctly classified 78.9% of cases, with a sensitivity of 83.3% and a specificity of 76.1%. The Hosmer–Lemeshow test did not indicate poor model fit, χ^2^(8) = 9.52, *p* = 0.301. These findings suggest that, in this dataset, professional exposure within the NBA, particularly previous NBA assistant coaching experience, was more clearly associated with NBA head coaching status than the demographic variables included in the model.

## 4 Discussion

The strongest finding of the present study was the association between previous NBA assistant coaching experience and NBA head coaching status.

NBA playing experience differed significantly across leagues and was also associated with NBA head coaching status, although this association was weaker than that of previous NBA assistant coaching experience. NBA head coaches showed the highest proportion of prior NBA playing experience, while international playing experience was most common among NBA G-League coaches. These findings are consistent with previous research suggesting that playing experience may help coaches better understand players' emotions, competitive stress, and team dynamics (11). Previous research has also suggested that some player-turned-coaches may demonstrate advantages in strategic decision-making (12). However, these experiences may be useful, but they do not automatically translate into elite coaching advancement (13).

This interpretation is also consistent with coaching-development literature emphasizing that coaches acquire expertise through multiple learning sources, including formal education, coaching experience, mentoring, interaction with other coaches and athletes, professional clinics, journals, and game film analysis (23–26). In the present study, the stronger association of NBA assistant coaching experience suggests that direct coaching-role experience within NBA organizations may be especially important for career advancement to NBA head coaching positions.

Some NBA-specific career pathway variables may provide additional context for interpreting the importance of previous NBA assistant coaching experience. For example, whether a coach became an NBA head coach within a franchise where he had previously worked may reflect internal promotion pathways, thanks to their deeper knowledge of the organization and their internal networks. Such variables were not included in the main regression model because they are only meaningful for coaches who had already entered the NBA coaching pathway. Therefore, they should be interpreted as exploratory career-pattern observations rather than independent predictors of becoming an NBA head coach.

Race/ethnicity distribution and university degree status did not show statistically significant differences across the three coaching groups in the current sample.

Taken together, the hypotheses were partially supported: NBA-related coaching experience showed the strongest association with NBA head coaching status, playing background showed a weaker but relevant association, while the demographic and educational variables included in the present analyses were not statistically significant.

Several limitations should be acknowledged. First, the study was cross-sectional and observational; therefore, the findings describe associations rather than causal effects. Second, the analytic sample was relatively small, particularly for multivariable regression, and the number of predictors was therefore restricted to reduce the risk of overfitting. Finally, the study focused on observable demographic, playing-career, and coaching-career characteristics, and did not capture internal decision-making processes, personal motivations, organizational hiring discussions, or subjective coaching experiences. Therefore, the findings should be interpreted as exploratory evidence of career-pathway associations rather than as proof of causal employment mechanisms.

## 5 Conclusion

The present study examined demographic, playing-career, and coaching-career characteristics associated with NBA head coaching status across NBA, NBA G-League, and NCAA Division I coaching environments. The findings suggest that NBA head coaching status was more strongly associated with NBA-related professional experience than with demographic background or formal educational variables.

Previous NBA assistant coaching experience showed the strongest association with NBA head coaching status. NBA playing experience was also statistically associated with this career outcome, although to a lesser extent. In contrast, age and White race/ethnicity were not statistically significant predictors in the final regression model. These findings should be interpreted as associations rather than causal effects.

Overall, the results suggest that advancement to NBA head coaching positions may be linked more closely to professional embeddedness and NBA-specific coaching experience than to demographic or educational characteristics alone. Future research should examine these career pathways using larger samples, longitudinal designs, and additional data on organizational decision-making and coaching development processes.

## Data Availability

The raw data supporting the conclusions of this article will be made available by the authors, without undue reservation.
